# Factors of mortality in patients with cardiac implantable electronic device: 5-year experience

**DOI:** 10.1590/1806-9282.20230998

**Published:** 2024-05-03

**Authors:** Kemal Göçer, Ahmet Çağrı Aykan, Akif Serhat Balcioğlu, Ekrem Aksu, Murat Kaniyolu, Musa Dağli, Naime Sıla Göçer

**Affiliations:** 1 Kahramanmaras Sutcu Imam University, Faculty of Medicine, Department of Cardiology – Kahramanmaraş, Turkey.; 2 Necip Fazıl City Hospital, Department of Infectious Diseases – Kahramanmaraş, Turkey.

**Keywords:** Defibrillators, implantable, Cardiac pacing, artificial, Hematoma, Mortality, Infection

## Abstract

**OBJECTIVE:**

The use of cardiac implantable electronic devices has increased in recent years. It has also brought some issues. Among these, the complications of cardiac implantable electronic devices infection and pocket hematoma are difficult to manage. It can be fatal with the contribution of patient-related risk factors. In this study, we aimed to find mortality rates in patients who developed cardiac implantable electronic devices infection and pocket hematoma over 5 years. We also investigated the risk factors affecting mortality in patients with cardiac implantable electronic devices.

**METHODS:**

A total of 288 cardiac implantable electronic devices patients were evaluated. Demographic details, history, and clinical data of all patients were recorded. Cardiac implantable electronic devices infection was defined according to the modified Duke criteria. The national registry was used to ascertain the mortality status of the patients. The patients were divided into two groups (exitus and survival groups). In addition, the pocket hematoma was defined as significant bleeding at the pocket site after cardiac implantable electronic devices placement.

**RESULTS:**

The cardiac implantable electronic devices infection was similar in both groups (p=0.919), and the pocket hematoma was higher in the exitus group (p=0.019). The exitus group had higher usage of P2Y12 inhibitors (p≤0.001) and novel oral anticoagulants (p=0.031). The Cox regression analysis, including mortality-related factors, revealed that renal failure is the most significant risk factor for mortality. Renal failure was linked to a 2.78-fold higher risk of death.

**CONCLUSION:**

No correlation was observed between cardiac implantable electronic devices infection and mortality, whereas pocket hematoma was associated with mortality. Furthermore, renal failure was the cause of the highest mortality rate in patients with cardiac implantable electronic devices.

## INTRODUCTION

Cardiac implantable electronic devices (CIED) have become more widespread recently. However, this rise has also brought about issues related to CIED, which can cause severe morbidity and mortality. The primary concerns with a CIED are infection and hematoma at the insertion site. Treating and managing CIED infection is a challenging task. Although its incidence is 0.13–19.9%, mortality due to CIED infection has been reported as 27–65%. CIED infections may result from patient, operator, or device-related factors^1^.

The development of a pocket hematoma often accompanies the CIED. A report indicates that a hematoma at the pocket site can act as a nutrient medium for microorganisms, multiplying the risk of CIED infection up to 20 times. Many factors, ranging from the use of antiplatelets and anticoagulants in the patients to surgical techniques, may contribute to the hematoma. It is important to determine the risk factors and establish treatment strategies to prevent CIED and pocket hematoma^2^. Research on this topic is still going on. The high death rates associated with CIED infections and the worries about the cost have prompted investigations into the research of risk factors. We examined cases of CIED infection and pocket site hematoma over a 5-year timeframe. In addition, we investigated patient-related risk factors that influence mortality in patients with CIED.

## METHODS

In this retrospective analysis, a total of 288 CIED patients who were admitted to our institution from January 2016 to December 2020 were evaluated. All procedures followed were in accordance with the ethical standards of the responsible committee on human experimentation (institutional and national) and with the Helsinki Declaration of 1975, as revised in 2008. Ethics committee approval has been granted from the ethics committee of Kahramanmaraş Sütçü İmam University Faculty of Medicine on November 1, 2022, with protocol number 02, and informed consent has been obtained from all participants.

Demographic details, history, and clinical data of all patients were recorded. The details of CIED [PPM (permanent pacemaker), ICD (intracardiac cardioverter defibrillator), cardiac resynchronization therapy (CRT)], the count of battery replacements, and the number of leads were registered. CIED infection was defined according to the modified Duke criteria. CIED infections developed within 60 days were categorized as early-stage CIED infection, whereas those developed after 60 days were considered late-stage CIED infection. Patients were segregated into two groups based on whether or not they developed a significant hematoma at the pocket site after the CIED. The significant pocket hematoma was identified as a swelling requiring drainage. Follow-up contacts were made by outpatient visits or telephone. Our hospital and national databases revealed the patient's admission information and whether they were alive.

The dates and causes of death of individuals were documented. The endpoint was accepted as all-cause death. In addition, the patients were divided into exitus and survival groups during the follow-up period from January 2016 to January 2023. Variables were compared between the two groups. The internationally accepted renal failure definition was a glomerular filtration rate below 60 mL/min.

### The procedure of the cardiac implantable electronic devices

Cardiac device implantation was performed in the catheter laboratory. A single gram of cefazolin antibiotic was given intravenously to all patients 30 min before the procedure. During the procedure, 10% povidone-iodine was administered to the patient's CIED pocket area. The skin was opened with cautery or a surgical scalpel. The axillary vein, or subclavian vein, was punctured. Direct skin-to-vein puncture was left to the operator's preference. A subcuticular suture was used for skin closure. Intravenous sedation and nasal oxygen were administered to all patients during the procedure. Oxygen saturation and blood pressure were monitored. All patients underwent anti-biotherapy for 1 week post-procedure.

### Statistical analysis

Data were analyzed using SPSS 22 (SPSS Inc., Chicago, IL, USA). The normality of the variables was tested according to the Kolmogorov-Smirnov test. The numerical data were given as the mean and standard deviation for the exitus and survival groups. The median interquartile range was used to express numerical data that were not normally distributed. Continuous variables were compared using an independent-sample t-test or a Mann-Whitney U test. Categorical variables were shown as percentages. The chi-square test was utilized to analyze categorical data. Kaplan-Meier analysis was performed for those who developed CIED infection and hematoma for survival analysis. The effects of variables on mortality were examined using Cox regression analysis. A p<0.05 was considered statistically significant.

## RESULTS

A total of 288 patients were included in the study, and 38.2% of these patients were females and 61.8% were males. The mean age of the patients with CIED was 65.64±15.24 years. Regarding CIED type, patients had 37.8% PPM, 37.2% ICD, and 25% CRT, respectively. The mean total lead count was 2.00±0.70. CIED patients were split into two groups: exitus and survival groups. Table 1 indicates the clinical and demographic comparisons between the two groups.

**Table 1:** Comparison of the demographic and clinical characteristics of the patients with cardiac implantable electronic devices who died and those who survived after 60 months of follow-up.

		Exitus group(n=166)	Survival group(n=122)	p-value
Age (years)		61.83±16.07	70.82±12.31	**<0.001**
Male, n (%)		98 (59)	80 (65.6)	0.259
Heart failure, n (%)		88 (53)	94 (77)	**<0.001**
Diabetes mellitus, n (%)		43 (25.9)	46 (37.7)	**0.032**
Atrial fibrillation, n (%)		40 (24.1)	31 (25.4)	0.798
Hypertension, n (%)		144 (86.7)	117 (95.9)	**0.008**
Malignancy, n (%)		4 (2.4)	4 (3.3)	0.657
CODP, n (%)		12 (7.2)	25 (20.5)	**0.001**
CAD, n (%)		119 (71.7)	103 (84.4)	**0.011**
Renal failure, n (%)		10 (6)	28 (23)	**<0.001**
Antiplatelet use, n (%)				
	ASA	110 (66.3)	92 (75.4)	0.094
	P2Y12 inhibitors	54 (32.5)	69 (56.6)	**<0.001**
Anticoagulation use, %				
	Warfarin	26 (15.7)	17 (13.9)	0.684
	NOAK	23 (13.9)	29 (23.8)	**0.031**
Steroid use, n (%)		3 (1.8)	3 (2.5)	0.702
Statin use, n (%)		95 (57.2)	66 (54.1)	0.597
Generator replacement, n (%)		23 (13.9)	16 (13.1)	0.856
Number of leads		1.95±0.66	2.08±0.75	0.113
Pocket hematoma, n (%)		1 (0.6)	6 (4.9)	**0.019**
CIED infection, n (%)		10 (6)	7 (5.7)	0.919

Bold indicates statistically significant p-value. ASA: acetylsalicylic acid; CAD: coronary artery disease; CIED: cardiac implantable electronic device; COPD: chronic obstructive pulmonary disease; NOAC: novel oral anticoagulant.

While CIED infection was similar in both groups (p=0.919), pocket hematoma was higher in the exitus group (p=0.019). The acetylsalicylic acid (ASA) (p=0.094) and Warfarin usage rates (p=0.684) were similar in both groups. Additionally, the percentages of ASA and warfarin usage were equal in both groups. In contrast, the exitus group had higher usage of P2Y12 inhibitors (p≤0.001) and novel oral anticoagulants (NOACs) (p=0.031).

**Figure 1:**
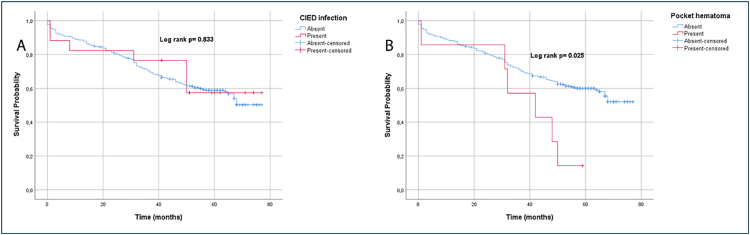
Survival tables of patients with pocket hematoma and cardiac implantable electronic device infection.

The CIED infection was detected in 17 (5.9%) patients in our study. No bacterial growth was detected in the blood culture. However, coagulase-negative *Staphylococcus* (CNS) had the highest incidence rate in the pocket site or device material swab. The microorganisms obtained from the pocket site or device material are elaborated. Early infection was observed in 11 (64.7%) patients and late infection in 6 (35.3%) patients. Six (100%) late infections were CNS-detected. Of those who developed CIED infections, 12 were removed by simple traction and five by the lead extraction procedure (using locking stylets and mechanical non-powered telescoping sheaths). Hematoma drainage was required in seven of the patients with CIED. In addition, the survival tables of those with pocket hematoma and CIED infections are illustrated in Figure 1.

The Cox regression analysis, including mortality-related factors, revealed that renal failure is the most significant risk factor for mortality. Renal failure was linked to a 2.78-fold higher risk of death. Diabetes mellitus (p=0.215) and hypertension (p=0.977) did not affect mortality. The remaining variables are denoted in Table 2.

**Table 2:** Cox regression analysis showing the effects of variables on mortality.

	B	OR	p-value	95%CI	
				Lower	Upper
Age	0.033	1.034	**<0.001**	1.017	1.051
Heart failure	0.715	2.044	**0.005**	1.240	3.369
Diabetes mellitus	0.247	1.281	0.215	0.867	1.892
Hypertension	0.015	1.015	0.977	0.369	2.793
COPD	0.804	2.234	**0.001**	1.417	3.522
CAD	-0.258	0.773	0.422	0.412	1.450
Renal failure	1.025	2.786	**<0.001**	1.787	4.345
Developing pocket hematoma	0.633	1.884	0.152	0.792	4.483
Developing CIED infection	-0.334	0.716	0.397	0.331	1.551
Use of P2Y12 inhibitors	-0.267	0.766	0.196	0.511	1.148
Use of NOAC	-0.172	0.842	0.441	0.543	1.305

Bold indicates statistically significant p-value. CAD: coronary artery disease; CIED: cardiac implantable electronic device; COPD: chronic obstructive pulmonary disease; NOAC: novel oral anticoagulant.

## DISCUSSION

In recent years, the prevalence of CIED infection has risen due to population growth, technological advancement, and the widespread usage of CIED in cardiac conditions. Examining indicators of CIED infection that result in severe illness and death can help us tackle the problem. Our goal with this study was to evaluate risk factors and introduce new treatments by analyzing the patients in our clinic who had CIED infection and hematoma over 5 years^3^.

The percentage of CIED infections in our study was 5.9%. Research conducted on 1326 CIED patients found that 2.4% had a CIED infection and 1.2% had a pocket hematoma^2^. Polyzos et al., conducted a meta-analysis of 60 studies, revealing a 1–1.3% CIED infection rate^4^. Mela et al., observed that only 1.2 out of 1700 CIED caused infection^5^. The incidence of infection was relatively low; however, the mortality rates were quite high. In a study, the 1-year mortality rate was 15–30% in patients who developed CIED infection^6^. In our study, the high rate of CIED infection may be due to poor sterilization conditions. However, due to the low mortality rate in the long-term follow-up of those who develop CIED infection, we can state that the infection was treated with an effective anti-biotherapy regimen without causing infective endocarditis.

Our findings indicate CNS as the predominant microorganism causing CIED infection. Results from a study conducted by Goya et al., showed that 181 CIED infections contained 30.1% CNS and 37.1% *S. aureus* ^7^. In a study by Bongiorni et al., on CIED, 69% of the microorganisms responsible for the infection were CNS, and 13.8% were *S. aureus* ^8^. Tarakji et al., reported that 44.4% of CIED infections were caused by CNS, 20.1% by methicillin-susceptible *S. aureus*, and 15.8% by methicillin-resistant *S. aureus* ^9^. Staff are commonly located in the skin flora. During the CIED procedure, it may penetrate through the open skin. Unsuitable antiseptic regulations augment the rate of staph infection. Administering beta-lactam antibiotics before and after the procedure decreases the risk of infection. Nevertheless, beta-lactam antibiotics are ineffective against methylene-resistant microorganisms present in 5–10%. Therefore, a single dose of vancomycin has been reported to be effective in prophylaxis for CIED infections^10^.

We found no relationship between CIED infection and the type of CIED. A retrospective meta-analysis study involving 78.267 French participants revealed that PPM, ICD, cardiac resynchronization therapy with a defibrillator (CRT-D), and cardiac resynchronization therapy with a pacemaker (CRT-P) infection rates were 0.5, 1.6, 1.6, and 1%, respectively. Of those who had a device replacement, the infection rates were 2.9, 2.9, 1.3, and 3.9% for PM, ICD, CRT-P, and CRT-D, respectively^11^. Harper et al., reported that the rate of CIED was between 0.3 and 1.1%, whereas the infection rate in those who experienced lead revision and upgrade was 2.1%^12^. Previous studies suggest the infection rate is linked to the number of leads, replacements, and device type. The magnitude of the device, the lead cable's thickness, and the presence of the coil may raise the risk of CIED infections. The vast majority of our patients had active fixation leads.

One of the most common complications of CIED is the presence of a hematoma. Patients with CIED are prescribed anticoagulants and antiaggregates due to their high risk of cardiovascular disease and atrial fibrillation. Previously, 0.9% of the participants in the unmedicated group had hematoma, while 5.5 and 5.6% of the ASA and NOAC groups had hematoma, respectively. In addition, with dual antiplatelet treatment, the incidence of hematoma increased up to five times^13^. Warfarin users have discontinued using heparin-bridging strategies because of the heightened danger of hemorrhage. An investigation revealed that the utilization of heparin for bridging treatment caused a 20% boost in a pocket hematoma. Therefore, uninterrupted NOAC treatments have been applied recently. Our research demonstrated that mortality rates were higher among NOAC and P2Y12 inhibitor users. The fact that P2Y12 inhibitors are more potent agents than ASA and the difficulty in finding the antidote for NOACs may have caused an increase in all-cause mortality^14^.

Cardiac conditions commonly co-occur with chronic diseases. There is a correlation between chronic diseases and CIED infections. A meta-analysis showed that diabetes mellitus, kidney disease, chronic obstructive pulmonary disease, malignancy, and heart failure were risk factors for CIED infection^15^. No relationship could be established between age and infection. A total of 2792 patients with CIED were analyzed by Qintar et al. Diabetes, young age, and heart failure were independent precursors of CIED infection^16^. Conflicting studies have been reported between age and CIED infection. Duval et al., described an increase in CIED infections in older individuals. Da Costa et al., revealed that CIED infections had increased among diabetes and dialysis patients^17^. The same study did not suggest that lack of antibiotic therapy, age, and cardiomyopathy were risk factors for CIED infection. A comparative analysis was not conducted since the number of CIED-infected patients was limited. In the CIED infection group, male gender, hypertension, coronary artery disease, and heart failure were particularly prevalent. Among those with CIED infection, renal failure was rare^18^.

Our study has some limitations. First, it included a retrospective collection of patients. Second, there were differences between CIED manufacturers in terms of battery sizes and thickness of leads. This can affect the CIED infection.

## CONCLUSION

No correlation was observed between CIED infection and mortality, whereas pocket hematoma was associated with mortality. Furthermore, renal failure was the cause of the highest mortality rate in patients with CIED.
